# Review of open-source software for developing heterogeneous data management systems for bioinformatics applications

**DOI:** 10.1093/bioadv/vbaf168

**Published:** 2025-07-18

**Authors:** Danilo Silva, Monika Moir, Marcel Dunaiski, Natalia Blanco, Fati Murtala-Ibrahim, Cheryl Baxter, Tulio de Oliveira, Joicymara S Xavier, The INFORM Africa Research Study Group, Christina Riley, Anna Winters, Vivek Naranbhai, Felix Made, Salim Abdool Karim, Kennedy Otwombe, Alash'le Abimiku, Sophia Osawe, James Onyemata, Patrick Dakum, Fati Murtala-Ibrahim, Nifarta Andrew, Aminu Musa, Tolulope Adenekan, Kenneth Ewerem, Victoria Etuk, Tulio de Oliveira, Cheryl Baxter, Eduan Wilkinson, Houriiyah Tegally, Jenicca Poongavanan, Michelle Parker, Danilo Silva, Joicymara S Xavier, Kristen A Stafford, Manhattan Charurat, Natalia Blanco, Timothy O'Connor, Meagan Fitzpatrick, Mohammad M Sajadi, Olanrewaju Lawal, Chenfeng Xiong, Weiyu Luo, Xin Wu

**Affiliations:** Centre for Epidemic Response and Innovation (CERI), School of Data Science and Computational Thinking, Stellenbosch University, Stellenbosch, 7602, South Africa; Computer Science Division, Department of Mathematical Sciences, Faculty of Science, Stellenbosch University, Stellenbosch, 7602, South Africa; Centre for Epidemic Response and Innovation (CERI), School of Data Science and Computational Thinking, Stellenbosch University, Stellenbosch, 7602, South Africa; Computer Science Division, Department of Mathematical Sciences, Faculty of Science, Stellenbosch University, Stellenbosch, 7602, South Africa; School for Data Science and Computational Thinking, Stellenbosch University, Stellenbosch, 7602, South Africa; School of Medicine, University of Maryland Baltimore, Baltimore, MD, 21201, United States; Department for Strategic Information, Institute of Human Virology (IHVN), Abuja, Federal Capital Territory, 900211, Nigeria; Centre for Epidemic Response and Innovation (CERI), School of Data Science and Computational Thinking, Stellenbosch University, Stellenbosch, 7602, South Africa; Centre for the AIDS Programme of Research in South Africa (CAPRISA), Durban, 4013, South Africa; Centre for Epidemic Response and Innovation (CERI), School of Data Science and Computational Thinking, Stellenbosch University, Stellenbosch, 7602, South Africa; Centre for the AIDS Programme of Research in South Africa (CAPRISA), Durban, 4013, South Africa; KwaZulu-Natal Research Innovation and Sequencing Platform (KRISP), Nelson R Mandela School of Medicine, University of KwaZulu-Natal, Durban, 4001, South Africa; Department of Global Health, University of Washington, Seattle, WA, 98195-7965, United States; Centre for Epidemic Response and Innovation (CERI), School of Data Science and Computational Thinking, Stellenbosch University, Stellenbosch, 7602, South Africa; Institute of Biological Sciences, Universidade Federal de Minas Gerais (UFMG), Belo Horizonte, 31270-901, Brazil; Computer Science Division, Instituto Tecnológico de Aeronáutica (ITA), São Paulo, 12228-900, Brazil

## Abstract

**Summary:**

In a world where data drive effective decision-making, bioinformatics and health science researchers often encounter difficulties managing data efficiently. In these fields, data are typically diverse in format and subject. Consequently, challenges in storing, tracking, and responsibly sharing valuable data have become increasingly evident over the past decades. To address the complexities, some approaches have leveraged standard strategies, such as using non-relational databases and data warehouses. However, these approaches often fall short in providing the flexibility and scalability required for complex projects. While the data lake paradigm has emerged to offer flexibility and handle large volumes of diverse data, it lacks robust data governance and organization. The data lakehouse is a new paradigm that combines the flexibility of a data lake with the governance of a data warehouse, offering a promising solution for managing heterogeneous data in bioinformatics. However, the lakehouse model remains unexplored in bioinformatics, with limited discussion in the current literature. In this study, we review strategies and tools for developing a data lakehouse infrastructure tailored to bioinformatics research. We summarize key concepts and assess available open-source and commercial solutions for managing data in bioinformatics.

**Availability and implementation:**

Not applicable.

## 1 Introduction

The world has faced massive data growth over the past three decades. Organizations that work with data must address big data management problems to make decisions and establish policies. While there is no consensus about the definition of big data, a good way to characterize it is according to the five V’s: volume, value, variety, velocity, and veracity (Campos *et al.* 2021). In bioinformatics and public health, the ability to store, process, and share data is vital to support research and produce relevant outcomes. The data produced and used across these areas can be structured, semi-structured, and unstructured, varying in type, format, and purpose. Given the rapid and steady production of large volumes of data, big data concepts such as the five V’s are highly applicable.

The Role of Data Streams in Informing Infection Dynamics in Africa (INFORM Africa) is a research hub focused on addressing public health issues in Africa and improving preparedness for future pandemics (Poongavanan *et al.* 2023). Its Data Management and Analysis Core (DMAC) collates data from partner institutions across multiple African countries. DMAC exemplifies an organization that works with bioinformatics and health data and struggles with data management as it aims to integrate data for its projects, which stems from various sources and includes genomic, human mobility, survey results, as well as clinical data (Poongavanan *et al.* 2023). DMAC’s data management challenges are closely related to the five V’s induced by heterogeneous big data. To maintain the projects’ various datasets and access requirements, challenges of heterogeneous, unorganized, standardless, and large data volumes must be addressed with appropriate big data concepts. Due to DMAC’s generic data management aims and challenges, their projects may serve as a good case study to identify user and system requirements.

Understanding data and its different structures is extremely important for data management projects in general, such as INFORM-Africa’s DMAC. In biological sciences, many data formats are used to organize and represent different organisms and phenomena. For example, the Sequence Alignment Map (SAM) is a semi-structured plain-text file format often used by genomics researchers. In contrast, the Binary Alignment Map (BAM) is a binary-compressed file format (SAM files’ binary version) that represents genome sequence alignments and maps (Li *et al.* 2009). Further examples are the Fast-All (FASTA) and Fast-Quality (FASTQ) file formats, which are also plain text, used to represent genomic data (Cock *et al.* 2010). The Variant Call Format (VCF) format is capable of encoding Deoxyribonucleic Acid (DNA) annotation data about gene variations (Danecek *et al.* 2011). Finally, generic structured and semi-structured formats such as Excel Spreadsheet (XLSX), Comma-Separated Values (CSV), JavaScript Object Notation (JSON), and eXtensible Markup Language (XML) are used to represent data and metadata in the field.

The use of relational databases was gradually adopted by the scientific community to address commonly observed data issues in bioinformatics. They organize data in table formats and provide the Structured Query Language (SQL) to search and manage data (Schulz *et al.* 2016). Although largely adopted, relational databases have shortcomings that can potentially hinder scientific projects when, for example, horizontal scalability (the capability to store and process data in a distributed manner) or flexible data schemas are required. Therefore, relational databases would not be able to address three of the important V’s of big data: large volume, value, and variety. Subsequently, non-relational databases (NoSQL) have emerged as an alternative solution to overcome some deficiencies of relational databases.

NoSQL databases are promising options for handling large and complex biological datasets (Deka 2017). They can store extremely large datasets in a distributed manner and can process unstructured and semi-structured data due to the different data formats they support. Consequently, NoSQL databases have better horizontal scalability than relational databases, a better option for handling large, heterogeneous, and dynamic datasets (Padhy *et al.* 2011, Mitreva and Kaloyanova 2013, de Brevern *et al.* 2015, Deka 2017, Fraczek and Plechawska-Wojcik 2017). This ability is vital for many bioinformatic applications that rely on multiple formats and schemas to represent genomic, geospatial, clinical, survey, and mobility data.

When dealing with large or heterogeneous data, paradigms such as data warehouses and data lakes play a key role in designing effective data management infrastructures (Nambiar and Mundra 2022). Data warehouses are data repositories that rely on relational-structured databases and enforce fixed data schemas to have good organization and governance, making them ideal for providing consistent and valuable data. They are typically domain-driven, designed to store structured data to address specific application purposes (Harby and Zulkernine 2022, Nambiar and Mundra 2022). This makes them valuable for conducting extensive data analyses to support decision-making processes. However, their schema enforcement can make them less flexible and inadequate at handling heterogeneous and unstructured data. Typically, data must go through ETL (Extract, Transform, and Load) pipelines to fit into the database’s schemas before it can be consumed by applications that expect reliable and organized data.

In bioinformatics and health-related areas, data warehouses are commonly implemented to manage large volumes of data in research and health systems; two examples are the Healthcare Research and Analytics Data Infrastructure Solution (HRADIS) and Health Level Seven (HL7). HRADIS was introduced as a design process to build data warehouses encompassing health research’s most commonly used databases (Ozaydin *et al.* 2020), which can be transferred to bioinformatics applications. HL7 was designed to be a set of standards to enable interoperability with data transfer among healthcare providers.

Alternatively, data lakes have emerged as an infrastructure design paradigm that comprises a large repository of data with no specific purpose other than data storage. Rather than storing structured and schema-based data, data lakes are designed to store any type of data (structured, semi-structured, and unstructured) in raw format (Terrizzano *et al.* 2015, Miloslavskaya and Tolstoy 2016). This allows data to be ingested into storage without prior processing, cleaning, and standardization, which offers more flexibility in managing heterogeneous data. Nevertheless, the lack of schema enforcement and robust governance makes it difficult to organize data and to ensure data quality, which may lead to difficulties when the data are indexed, searched, or analyzed.

A new and promising approach for heterogeneous data management, known as data lakehouses, has recently emerged (Zaharia *et al.* 2021). This approach overcomes the deficiencies of data lakes and warehouses mentioned earlier while unifying their best strengths. Additionally, it addresses the five V’s of big data challenges caused by high data volume, velocity, variety, value, and veracity (Zaharia *et al.* 2021). Its main principles can be summarized as scalability (ability to allocate more computational resources as the data volume grows), flexibility (support multiple data types and formats), layered architecture (storage layer, ingestion layer, analytical layer), data governance (valuable data, data catalog, access control, data versioning), and interoperability (can be accessed by other applications). Due to its flexibility and organization, it is a strong design candidate to address the heterogeneous data issues in bioinformatics and can be adopted by research groups to efficiently manage their data (Begoli *et al.* 2021).

Despite the availability of many big data management solutions on the market, their utility has not been fully explored in bioinformatics (Benjamin *et al.* 2022). Data lake and Warehouse solutions, built by researchers and organizations, are either tailored to specific purposes (Miloslavskaya and Tolstoy 2016, Nambiar and Mundra 2022, Visweswaran *et al.* 2022) or do not have their design process publicly available, such as paid solutions like Snowflake (Snowflake 2024) and Databricks (Databricks 2024). This makes it difficult to replicate those solutions. Regarding data lakehouses, the lack of literature describing lakehouses systems for bioinformatics makes it an underexplored solution. Without well-documented processes of how data management architectures are built for any bioinformatics-related data, going forward, it is difficult to establish standards and best practices for building this type of infrastructure.

Regardless of the approach adopted for data management tasks, the scientific community has worked on setting up guidelines for standardizing data management tasks. The Findability, Accessibility, Interoperability, and Reproducibility (FAIR) principles have emerged as a popular guideline for the development of digital resources such as datasets, source code, workflows, and research software packages for data management applications (Wilkinson *et al.* 2016, 2017, Tang 2020). These principles aim to make resources easily findable and accessible to the target audience. Furthermore, it aims to democratize the use and replication of digital resources in scientific communities by making them interoperable and reusable. These principles are considered important to ensure that high data management standards are met in research applications (Tang 2020).

In this paper, we review current models and approaches for building data management infrastructure for bioinformatics applications. We also present an overview of the available open-source tools and software packages with which data management infrastructure may be deployed. Lastly, we assess their usability to fulfill a set of system requirements that we define as integral to a solution, in addition to their compliance level with the FAIR principles. In addition, we assess requirements commonly mentioned in the literature, such as scalability, ability to integrate with other tools, data security mechanisms, and ease of integration.

## 2 Methods

Our first objective was to identify the commonly used approaches, open-source tools, and off-the-shelf solutions that can be used to build data management infrastructure to store heterogeneous data arising from bioinformatics research. To identify relevant studies in the field, we conducted a manual search on PubMed, Science Direct by Elsevier, and Google Scholar publication databases. We obtained an initial reference list using broad keywords such as “data lakes in bioinformatics,” “genomic data lakehouses,” “data warehouses,” “genomic data lakes,” “data lake infrastructures for biological data,” “biological data lakes,” “biological data warehouses,” “big data systems,” “big data management,” “biological data management,” and “medical data management systems.” Furthermore, we used the relevant tools and concepts found in the initial reference list as keywords to manually search for further relevant papers and concepts. Data management tools and approaches, studies not written in English, or published before 2010, were excluded from the reference list.

**Figure 1.**
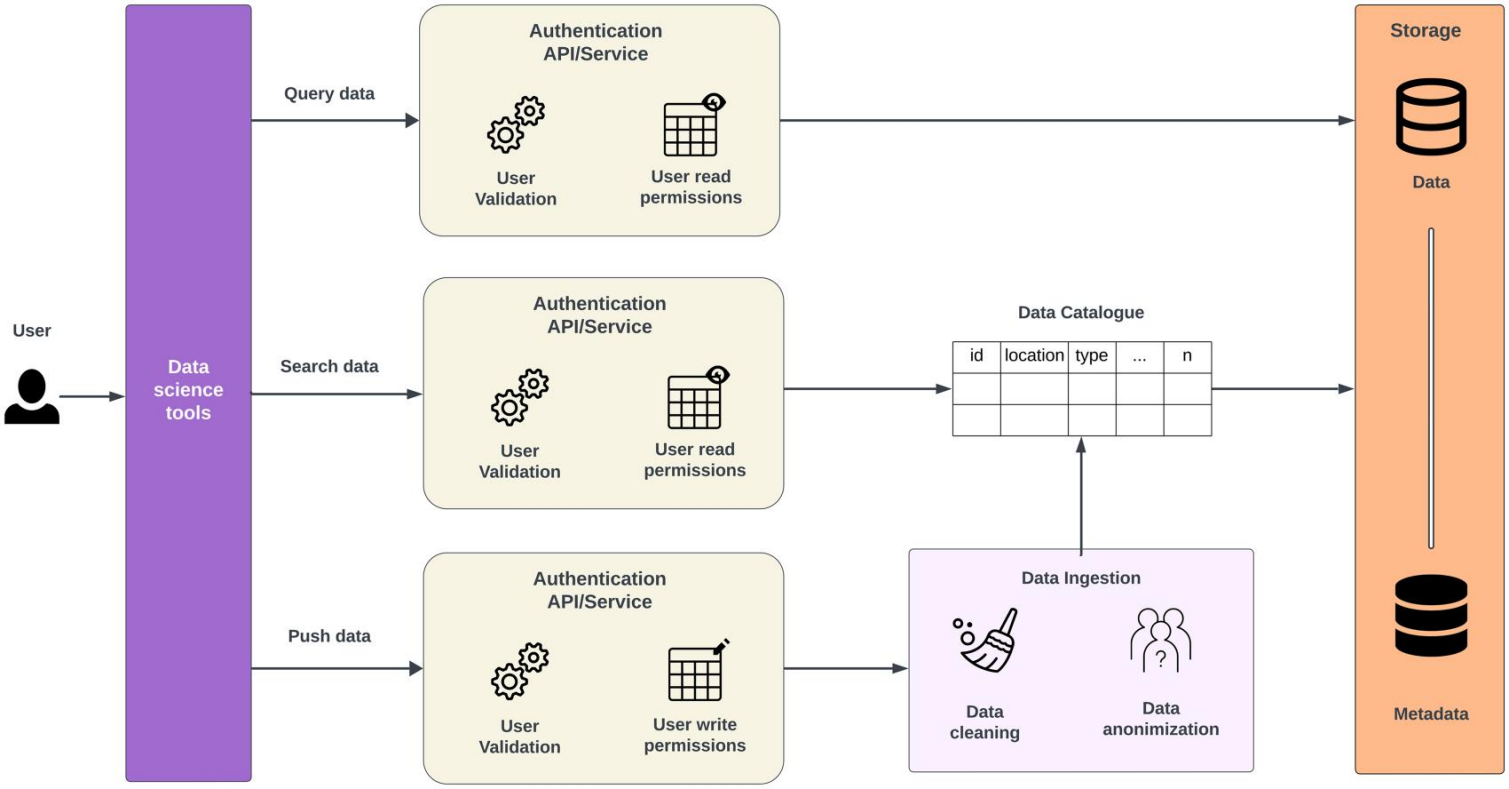
Identified user perspective based on the INFORM Africa Research Hubs projects. User requirements fall into three main categories: (1) interacting with infrastructure using data science tools for querying, inserting, and searching data; (2) user authentication and permissions management; and (3) managing data repositories through a data catalog, including data cleaning and anonymization.

**Figure 2.**
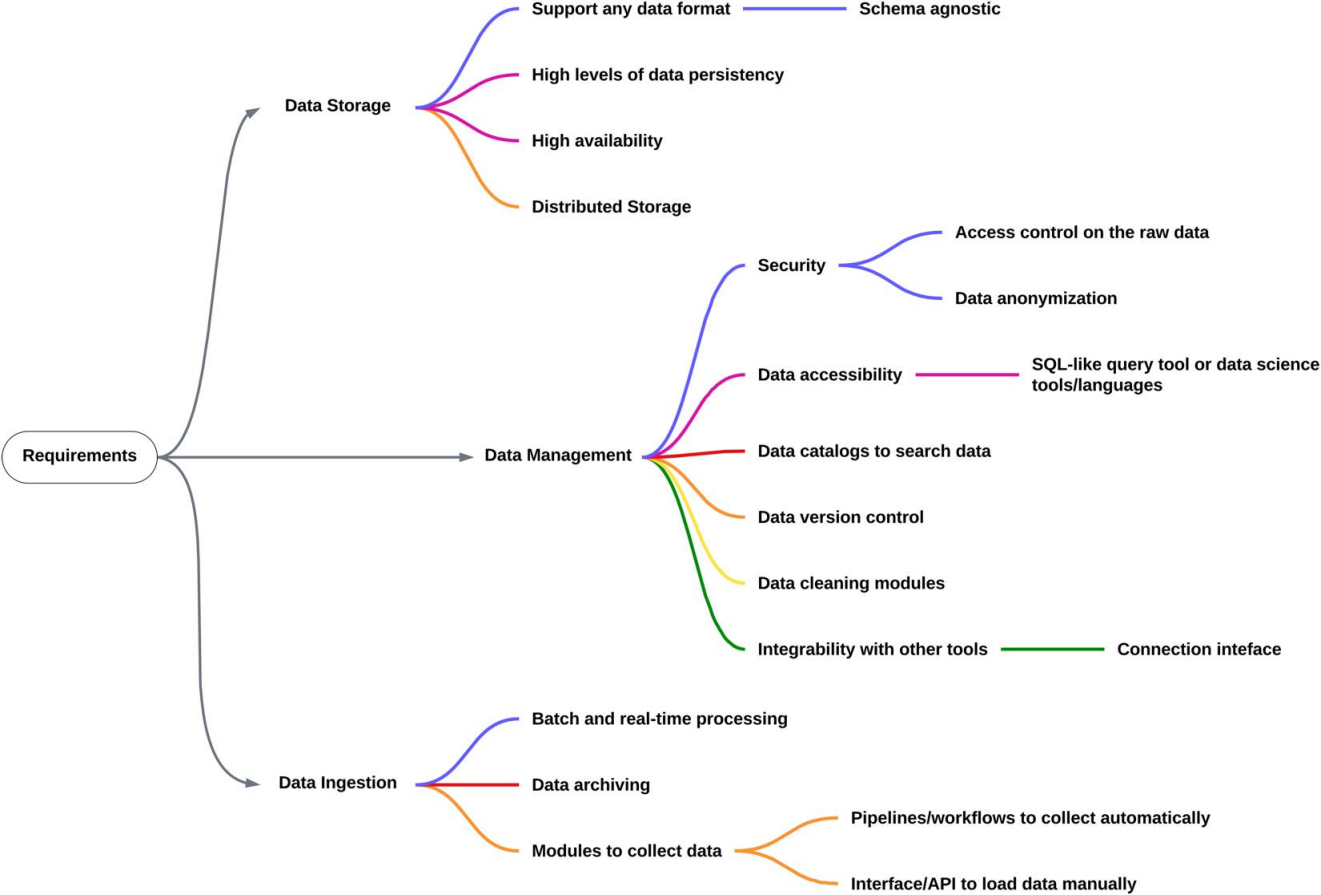
Data storage, ingestion, and management software requirements. Support for heterogeneous data enables schema-agnostic storage. Strong data persistence is vital to ensure that all ingested and processed data remains intact, while high availability is required to keep the infrastructure continuously accessible. Distributed storage is necessary to enable horizontal scaling as the volume of data grows. For our use case, the system must provide data ingestion functionalities to facilitate automated data collection through batch processing, as well as an interface or API to enable manual ingestion of data. Real-time and stream processing are important for certain use cases, while data archiving is a requirement to ensure optimal data storage. The management requirements outline the core functionalities the infrastructure must provide, including the organization and optimization of data access control, security, cataloging, integrability, and other important features for our use case.

The second objective was to identify and describe commonly occurring data management challenges within the context of bioinformatic research applications and to define the requirements to address those challenges. Having clear requirements was necessary to narrow down the list of relevant tools and approaches to address data management problems. For this goal, we utilized INFORM-Africa’s DMAC core as a use case. By addressing the big data and metadata challenges and requirements encountered by DMAC (Poongavanan *et al.* 2023), based on the five V’s of big data, we synthesized user requirements to guide our review process, and to guide the search for tools that best suited for our user needs (Fig. 1). Based on the user requirements (Supplementary Table S1, available as supplementary data at *Bioinformatics Advances* online), we identified important software requirements that a data management infrastructure must meet in order to address most of the identified data management challenges (Supplementary Table S2, available as supplementary data at *Bioinformatics Advances* online). All the tools and solutions presented in the Results section were assessed according to the features defined in the software requirements for data storage, ingestion, and management (Fig. 2).

For the data storage and management design considerations, one of the main user requirements is to support any data format. This requirement necessitates schema flexibility, which in turn means that the underlying data storage must be schema-less, which also allows future changes in data organization. In addition to flexibility, it is important to have durability to make sure that changes in the data storage will be performed only after the transactions are done, avoiding erroneous data being stored. Lastly, fault tolerance is vital to ensure that the system remains robust against unpredictable faults and provides high data availability.

Pertaining to the processing requirements, we focused on tools with batch processing capabilities since real-time data processing is not strictly necessary based on the user requirements, but rather an auxiliary feature. Furthermore, to allow data exploration, it is crucial to have a query language catered to data catalogs. Query languages such as SQL are useful for easy data access and are widely accepted by the scientific community. Data version control is also required to keep track of all changes in the data as well as associated metadata.

Horizontal scalability is essential to ensure long-term sustainability and accommodate future data needs, making distributed storage and processing a crucial system requirement. High data availability is also an essential system requirement, which necessitates that the tool guarantees that enough independent nodes are running with replicated data. Lastly, best practices for user management and access control must be implemented to ensure data security.

## 3 Data lakehouse and FAIR principles

**Figure 3.**
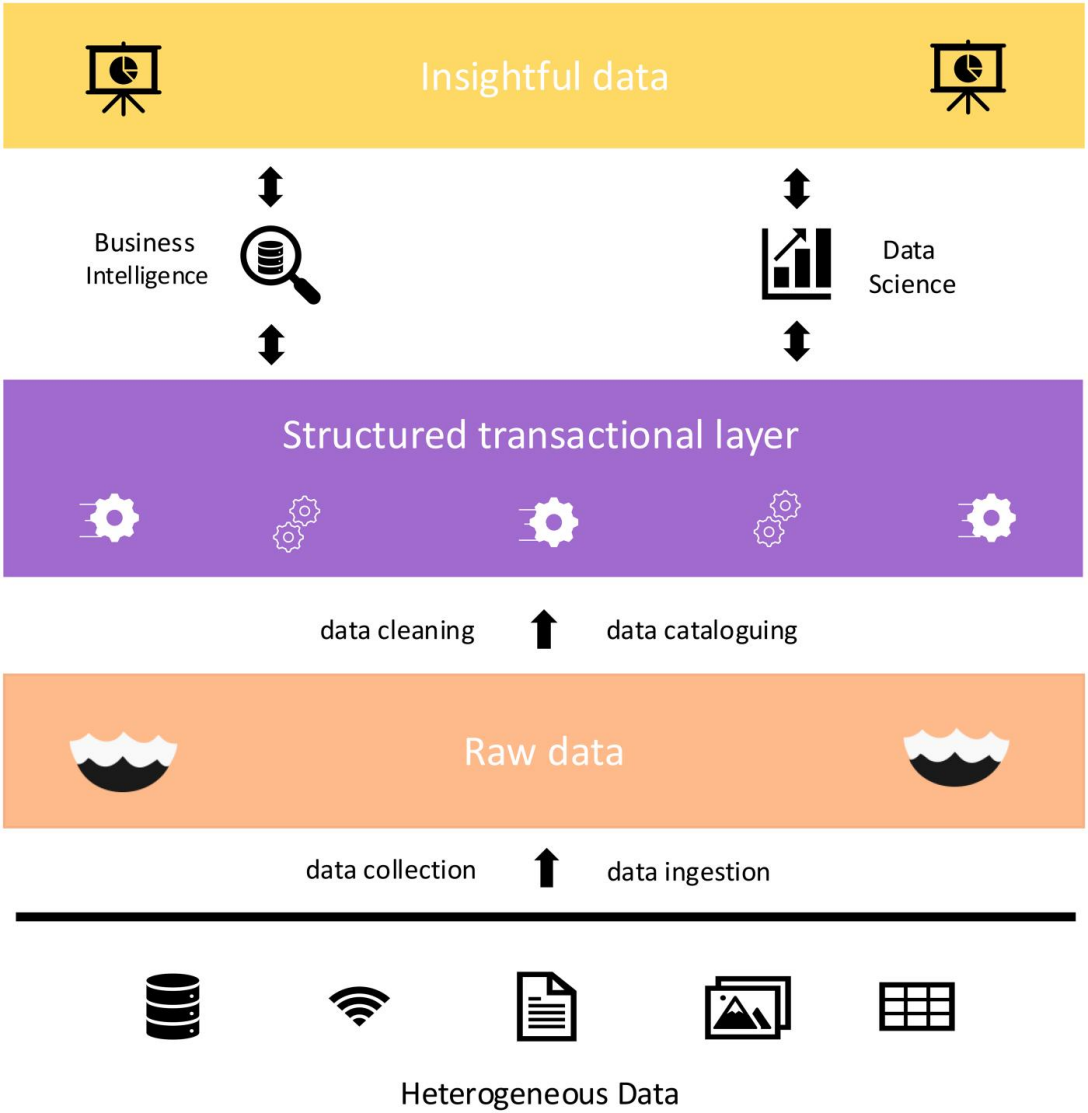
An example of a data lakehouse structure. The architecture of a data lakehouse comprises three core layers: the bottom layer with the raw data storage, the middle layer comprising structured data, organized to facilitate queries and online analytical processing (OLAP), and the top layer (data access) that consists of highly processed data to support data science and business intelligence applications.

An effective data lakehouse infrastructure must be architected with three main layers: data ingestion, data storage for raw data, and data access (ie, the transactional layer), aligned with the system requirements defined previously (Fig. 3). The ingestion layer must be capable of accepting, pre-processing, and cleaning, as well as cataloging datasets. The storage layer must be capable of storing raw data and processed data. Lastly, the transactional layer must be capable of storing tabular and structured data for online analytical processing tasks (OLAP); an access control layer; and a serving layer for the users to be able to consume data from the transactional layer. OLAP capabilities are crucial to enable information extraction from data. For example, they have supported researchers, epidemiologists, and policymakers in understanding diseases and pathogenic organisms during outbreaks (Leung *et al.* 2020). OLAP tools can also improve effective data access when combined with guidelines such as the FAIR principles.

Despite the aspirational concept, the FAIR principles are not strictly defined. No rules are enforced, but rather a set of attributes and behaviors to guide scientists and developers to achieve the FAIR goals (Wilkinson *et al.* 2017). As these principles have been adopted in several studies related to research data (Lamprecht *et al.* 2020, Tang 2020, Barker *et al.* 2022), they may be used as guidelines to build effective data management architectures. The FAIR for Research Software (FAIR4RS) is defined as adaptations of the original FAIR principles for developing research software (Barker *et al.* 2022). In opposition to the FAIR principles, the FAIR4RS principles look more objectively defined and can be applied to make new research software findable, accessible, interoperable, and reusable.

Strategies such as defining a unique software name, including descriptive metadata, and using public software repositories like Docker Hub and GitHub will not only help ensure open-source access but also support the findability and accessibility principles from FAIR4RS. Additionally, documenting the software with clear language and detailed instructions, along with a free software license, is essential for complying with the reproducibility principle. Finally, implementing public Application Programming Interfaces (APIs) and code libraries for other users and programs to interact with will ensure its interoperability.

Despite promoting good governance, the adoption of FAIR principles does not guarantee data security. In bioinformatics research, data security has emerged as a critical concern. While unrestricted access to raw data could ideally benefit and accelerate research, health data are often sensitive as they are linked to patients and their private information. For example, the use of data for genomic studies collected from human subjects almost always requires their consent and is often confidential.

There are typically four main security concerns when handling data from bioinformatic studies: ethical considerations, data privacy, secure storage, and data custody (Ferguson 2021). To facilitate data exchange, partnering organizations typically have data-sharing agreements in place; however, the data are rarely made publicly available or centralized in a data pool environment (Rehm *et al.* 2021). In addition, data sharing across national and institutional borders can also raise concerns as it might have to comply with different data regulations. Therefore, implementing appropriate data security requirements and access policies requires solutions beyond simple user authentication control (Thorogood *et al.* 2021).

To conclude, implementing data management architectures following FAIR’s guidelines depends heavily on domain-specific requirements (Begoli *et al.* 2021). For our needs, ensuring reusability and interoperability is vital, which can be achieved through effective metadata management. For enhanced data governance capabilities, .

## 4 Designing data architectures for bioinformatics applications

Designing a data lakehouse is a challenging task, especially for research applications, due to the lack of clear references and guidelines. To address this gap, studies of data lakes can be used as a foundation to build the storage and ingestion layer of the lakehouse, while best practices from data warehouse studies can be used to build the data access layer (transactional layer with data extraction and transformation). This design strategy allows for a logical separation of architecture, while still preserving the identity of a lakehouse paradigm (Harby and Zulkernine 2022). However, it is difficult to find tools that natively offer all data lakehouse features (ie, features to fulfill data ingestion, storage, and access requirements). As a result, selecting a suite of tools to cover different lakehouse functionalities and integrating them into a cohesive system is often necessary.

Using the software requirements list as selection criteria (Fig. 2), we identified promising software tools with functional features that fulfill the desired system capabilities and can be incorporated into a typical data management infrastructure for bioinformatics and health science applications. In the sections below, we present these tools in three categories: storage tools, ingestion tools, and metadata management tools.

## 5 Storage tools

In this section, we explain different data storage options, such as databases, object storage, file systems, and data warehouses. We also highlight their relevant features that meet the software requirements for building an effective data management solution. For the storage tools, we analyzed the type of storage, the ability to accept multiple data formats, support flexible schemas, durability, fault tolerance, and distributed storage. Table 1 summarizes these tools.

**Table 1.** List of storage tools and their relevant features.

Name	Reference	Type	Multiple data formats	Schema flexibility	Durability	Fault tolerant	Distributed storage
Amazon S3/GCS	(Amazon S3 2024)	Object Storage	Yes	Schema-less	High	Yes	Yes
Apache HBase	(Apache HBase 2024)	NoSQL tabular database	No	High	High	Yes	Yes
Apache Hive	(Apache Hive 2024)	Data warehouse system	No	High	Low	Yes	Yes
Ceph	(Ceph Foundation 2024)	File System, block/object Storage	Yes	Schema-less	High	Yes	Yes
GlusterFS	(GlusterFS 2024)	File System	Yes	Schema-less	Low	Yes	Yes
Hadoop HDFS	(HDFS 2024)	File System	Yes	Schema-less	High	Yes	Yes
MongoDB	(MongoDB 2024)	NoSQL document database	No	High	Low	No	Yes
OpenStack Swift	(OpenStack Swift 2024)	Object Storage	Yes	Schema-less	High	Yes	Yes

### 5.1 Databases

Non-relational databases can support the development of data management infrastructure and software for research. MongoDB is a non-relational database that has been widely adopted by the research community due to its flexibility and good performance for large data when compared to other NoSQL databases (Messaoudi *et al.* 2017). It is a document-oriented database that stores data in JSON records. For instance, “Global.Health” (an initiative producing software and frameworks to support infectious diseases research for public health applications) recently built a scalable platform for pandemic data integration and analytics using MongoDB as the main data repository, demonstrating that this database system can be effective in building applications to support research in this field (Global.Health 2024).

Apache HBase, an alternative NoSQL database, can also be used to support large-scale data storage in bioinformatics applications (Taylor 2010, Mrozek *et al.* 2016, Sebaa *et al.* 2018). It offers distributed storage, fault tolerance, and high scalability (Vora 2011). As part of the Apache ecosystem, it integrates with Apache HDFS and other tools designed for data management. Despite being schemeless, its column-oriented characteristics allow data to be stored in a tabular format similar to SQL databases. However, like other tools in the Hadoop ecosystem, it requires technical expertise for setup and maintenance.

### 5.2 File systems

The Hadoop File System (HDFS) has been available for almost 20 years, and it is the most adopted and reliable tool for building large data management infrastructures, given its capabilities to store unstructured data (Hambardzumyan *et al.* 2022) and to integrate with additional tools from the Apache Foundation’s data software suite (Sebaa *et al.* 2018). Therefore, HDFS offers high interoperability and can be used to build more complex systems, while also satisfying all the system requirements defined for this study (supporting multiple data formats, schema flexibility, durability, fault-tolerance, and distributed storage).

Apache Hadoop can easily scale to handle massive volumes of bioinformatics research data and can be used to build toolkits to analyze large-scale data in automated systems. It can be configured to serve either as a data lake or a lakehouse (Nordberg *et al.* 2013, O’Driscoll *et al.* 2013, Ferraro Petrillo *et al.* 2021, Zaharia *et al.* 2021, Couto *et al.* 2022). Nevertheless, there are newer solutions that can also handle heterogeneous data effectively. We identified GlusterFS and CephFS as alternative file system solutions to HDFS due to similar feature sets. For example, GlusterFS has been shown as a high-performance file system for genomic data management (Heath *et al.* 2014), as used by Bionimbus, an open-source cloud computing platform for managing large genomic datasets. One of Ceph’s storage types, CephFS, has not been thoroughly explored in the scientific literature for similar data management use cases. However, it is frequently used by computing systems that handle distributed data processing and storage, such as High-Performance Computing (HPC) platforms and cloud computing environments (Shah *et al.* 2022).

In terms of versatility, the Ceph stack appears to be the most promising option at first glance. It provides interfaces for block storage (RBD), object storage (RADOS), and a complete file system (CephFS), making it a comprehensive solution for data storage scenarios. CephFS’s fault-tolerance and data recovery mechanisms support automatic recovery, but they require a solid knowledge of CephFS’s internal architecture in cases where the data pools are damaged. Therefore, data loss might still occur if the recovery mechanisms are not used correctly (Shah *et al.* 2022). For instance, GlusterFS is the only file system listed that does not provide automatic recovery during failures (Acquaviva *et al.* 2018).

HDFS’s fault-tolerance and data recovery mechanisms are more robust and reliable, even in the presence of failures. The only type of failure that requires manual intervention is a NameNode (main node) failure. However, it does not result in data loss, as the data are stored in replicas across different DataNodes, and the HDFS client implements checksum routines to guarantee data consistency (Shah *et al.* 2022). Additionally, HDFS offers a code library that enables users to easily interact with the file system transparently via REST APIs.

In terms of benchmark tests, HDFS outperformed GlusterFS on reading and writing operations, although CephFS presented the best performance overall (reading and writing operations), according to a benchmark study published in 2018 (Acquaviva *et al.* 2018). In a benchmark study, carried with RAID-enabled storage disk arrays, published in 2021, CephFS outperformed GlusterFS at sequential and random read operations, while showing slower performance at writing operations in general (for random disk writing, CephFS outperforms GlusterFS when configured with a larger block spaces) (Lee *et al.* 2021).

### 5.3 Object storage

Object storage is also a popular approach for building heterogeneous data storage systems. OpenStack Swift is one of the most popular open-source object storage solutions (Chekam *et al.* 2016). It also offers distributed storage, scalability, and it was designed to be durable. Additionally, it provides an API to interact with external applications, making it highly interoperable. However, it is necessary to set up the OpenStack for the number of replicas per object/record saved in the database and the number of objects stored in each node (Chekam *et al.* 2016). Technical expertise is needed to set up an OpenStack Swift cluster effectively; otherwise, it will result in an object sync/replication problem. On the other hand, the Ceph stack also offers a stable and distributed object storage system suited for distributed data and high-throughput (lower latency) applications (Acquaviva *et al.* 2018). Ceph’s object storage is unified with Ceph’s block storage and file system, ensuring strong data consistency and fault-tolerance. Furthermore, Ceph object storage is claimed to be compatible with S3 and Swift API, allowing applications to connect to it using either Swift or S3 API, according to the available documentation (Ceph Foundation 2024).

Over the past decade, cloud services such as Amazon S3 and Google Cloud Storage (GCS) have transitioned from Hadoop File System to object storage solutions to build data lake infrastructures (Zaharia *et al.* 2021). This is mostly due to lower costs, high performance, and ease of maintenance (since they are fully managed). Although cloud storage services such as GCS and S3 are not open source like Swift and Ceph’s object storage, they have frequently been adopted by organizations and companies to replace the Hadoop File System when designing big data applications. Of the various cloud services, S3 has the lowest cost and offers nearly twice the performance compared to HDFS alternatives (Harby and Zulkernine 2022). Thus, using cloud services such as S3 would give applications the same level of computational power as an HDFS solution, but dismissing the need for technical skills and maintenance that solutions like Ceph and Swift require.

### 5.4 Data warehousing

Hive is a data warehousing platform that runs on top of HDFS and can provide data in tabular format through SQL-like query languages (Apache Hive 2024). It may be an ideal candidate to implement the transactional layer on top of the data lake storage. It can be configured to assure high availability and offers high durability (the ability to persist changes if transactions are not completed). Since it was built to run on top of the Apache Hadoop File System, it is strongly dependent on the use of HDFS to effectively work with data. Furthermore, even though it exposes SQL-like syntax to query data which makes the data trivially searchable, Apache Hive present itself as a difficult tool to integrate with other data science tools out of the box. We recommend Apache Hive only in situations where HDFS is the main data storage system in the infrastructure, since it may otherwise be difficult to build, integrate, and manage Apache Hive with other tools.

## 6 Data ingestion tools

Data ingestion and processing capabilities are imperative to ensure the effectiveness of a data lakehouse infrastructure. Ideally, data should not be highly processed before it is loaded into the architecture’s bottom layer (data lake layer), but the lack of cleaned metadata and data management routines can transform the data lake into an unorganized, difficult to use, and unmanageable data repository that is often referred to as a data swamp (Che and Duan 2020, Begoli *et al.* 2021, Sawadogo and Darmont 2021, Hambardzumyan *et al.* 2022, Harby and Zulkernine 2022, Nambiar and Mundra 2022). Therefore, metadata cleaning and processing is necessary to optimize data organization, consistency, and reduce the risk of having a swamp with redundant, unorganized, corrupted, or unfindable data. Furthermore, batch and stream (real-time) processing, in a distributed manner, is also imperative to guarantee scalability.

Our specific use case for this study does not require extensive stream processing compared to other real-world applications such as social media and IoT applications in health, geolocation, or finance. Despite that, stream processing is an important feature to make sure that the architecture will be able to handle real-time data as the project and the use cases expand. Here, we discuss the features of each data processing tool displayed in Table 2 and their potential to be part of the data ingestion layer. Features such as the ability to support batch processing, stream processing, and data compression were analyzed.

**Table 2.** List of ingestion and processing tools and their relevant features.

Name	Reference	Batch processing	Real-time processing	Compresses data
Apache Flink	(Apache Flink 2024)	Yes	No	No
Apache Kafka	(Apache Kafka 2024)	No	Yes	No
Apache NiFi	(Apache Nifi 2024)	Yes	Yes	Yes
Apache Spark	(Apache Spark 2024)	Yes	No	Yes
Apache Sqoop	(Apache Sqoop 2024)	Yes	No	No
Apache Storm	(Apache Storm 2024)	No	Yes	No

Open-source solutions such as Apache Spark, Storm, Flink, Sqoop, NiFi, and Kafka are widely adopted for handling and ingesting high volumes of data (Harby and Zulkernine 2022). They are highly interoperable with Apache HDFS and can also be integrated with databases and cloud storage services. Although they serve similar purposes, each one of them offers distinct features that can be used in different scenarios.

Spark is a general-purpose tool for processing large-scale data in a distributed manner. It can be used for stream processing, but it emphasizes batch processing and analytics capabilities (Apache Spark 2024). Likewise, Apache Storm is a low-latency, distributed, and fault-tolerant processing tool that is best suited for stream processing and real-time applications (Apache Storm 2024). Since it offers good capabilities for real-time processing, Storm lacks comprehensive batch processing and event-driven processing. Unlike Spark and Storm, Flink was designed to efficiently process both batch and stream data, supporting real-time processing with low latency and fault tolerance. In addition, it offers event-time tracking, which records all processed events as they are triggered by the data source (Apache Spark 2024).

Sqoop is mainly designed for data ingestion. It features distributed bulk data transfers from relational databases to HDFS and can be very useful when access to relational databases is provided (Apache Sqoop 2024). Similarly, Apache NiFi is a data flow management tool used for data ingestion. It enables the integration of various processing tools for sequential data transfer and ETL operations (Apache Nifi 2024). Among the other processing tools presented in this study, it is the only one that offers a user interface for visual management of data pipelines.

The use of Spark, Storm, Flink, and Kafka are largely explored in studies focusing on the design of big data processing systems for biological applications (Zhou *et al.* 2017, Lawlor *et al.* 2018, Baig *et al.* 2021, Htut and Aswakul 2022, Matteussi *et al.* 2022, Bartolini and Patella 2023). These tools can be combined with others scalable data repositories, such as HDFS and HBase, to build a fully self-managed data lake or lakehouse infrastructure. In particular, Apache Spark has been implemented in many use cases to process and reduce large volumes of DNA sequence data for analytical purposes (Wiewiórka *et al.* 2014). The study presented in Mrozek *et al.* (2014), Mrozek *et al.* (2016), and Couto *et al.* (2022) showcased the versatility of Hadoop for bioinformatics applications and the possibility to create a model to integrate heterogeneous data from bioinformatics using data lake concepts. They used Nifi for data ingestion and Python scripts to process data.

It appears that the Apache tools are seamlessly interoperable within the named Hadoop Ecosystem, but not all of them can be easily integrated with third-party solutions. In addition to offering strong performance for batch and stream data processing (Harby and Zulkernine 2022), Spark and Kafka can still be easily integrated with external software and programming languages such as Python. This enables the development of customized data ingestion strategies in a data lakehouse infrastructure.

## 7 Data and metadata management tools

In addition to storing and processing data effectively, good data and metadata management rules often lead to better data handling practices, higher data quality, improved data security, and better overall data organization. In this section, we dive into the specificities of the data management tools listed in Table 3. We analyzed features such as the support for query engines, data cleaning, cataloging, version control, user access control, and interoperability/integration with external tools to fulfill the system requirements identified for this study.

**Table 3.** Classification of data and metadata management tools and their relevant features.

Name	Reference	Query engine	Data cleaning	Cataloging	Version control	User/Data access control	Integrates to external tools
Apache Atlas	(Apache Atlas 2024)	Yes	No	Yes	No	No	Yes
Apache Ranger	(Apache Ranger 2024)	No	No	No	No	Yes	Yes
Delta Lake	(Delta Lake 2024)	Yes	No	Yes	Yes	No	Yes
Dremio lakehouse	(Dremio 2024)	Yes	No	No	No	Yes	Yes
GA4GH Data Connect	(Global Alliance for Genomics & Health 2024a)	Yes	No	Yes	No	No	No
GA4GH Passport Broker	(Global Alliance for Genomics & Health 2024b)	No	No	No	No	No	Yes
LakeFS	(Treeverse 2024)	Yes	No	No	Yes	Yes	Yes

Part of the solutions listed here are not widely cited in the literature, although they are popular in industry and can be used to fulfill the needs for good data governance. Except for the tools provided by the GA4GH group and the Delta Lake solution (Begoli *et al.* 2021, Zaharia *et al.* 2021), the identified tools in our list have not been widely studied and reviewed in the literature. Nonetheless, they are commonly used and contain features that address all our identified user and system requirements.

Atlas and Ranger are solutions based on Apache products that can integrate not only with the broader Apache Hadoop ecosystem, but also with some of the other commonly used data management and storage tools. Atlas supports data catalog management as it centralizes all the information about data assets distributed across the Hadoop Ecosystem, including data lineage and metadata (Tall and Zou 2023).

Ranger is a data security and governance solution built for the Hadoop Ecosystem (Mishachandar *et al.* 2021). It centralizes user access management, making sure that only authorized users have access to the right data (Gupta *et al.* 2017). For Lakehouse systems implemented with Hadoop HDFS, Apache Ranger combined with external authentication mechanisms, such as Kerberos, can be considered if strong application and user access control is required. Kerberos is a network authentication protocol designed to guarantee robust authentication for client-server applications by implementing secret-key cryptography (Mishachandar *et al.* 2021), considered a secure method for data transmission, as it never exposes passwords in an unencrypted form.

Delta Lake is a storage framework that can be used to enable the creation of a lakehouse infrastructure. It does not store the raw data itself but allows users to integrate raw data repositories (such as Hadoop HDFS, Google Cloud Storage, and Amazon S3) with data science tools for querying and processing data (such as Python, Apache Spark, and Apache Hive). Based on its documentation, Delta Lake is a framework that can be executed on top of the data lake storage itself. It leverages the Apache Spark Application Programming Interface (API) to process data and makes use of the Apache Iceberg format to store metadata, data catalogs, and data management records in table formats (Delta Lake 2024). Thus, Delta Lake provides a transactional storage layer that is structured and facilitates metadata management, and it is compatible and can be controlled using Apache Spark.

Dremio Lakehouse is a query engine that runs on top of data lakes, bringing data warehouse capabilities and facilitating data queries (Schneider *et al.* 2023). Dremio comes in two versions: the community open-source edition and the fully managed cloud edition. It can be used on top of Delta Lake to provide an analytical layer between the lakehouse and the end user.

The GA4GH toolkit enforces technical standards for responsible data sharing and thereby facilitates easier interoperability amongst research applications in bioinformatics. Its Data Connect and the Passport Broker services may be used to make our data infrastructures broader by integrating them into the storage layer.

Data Connect is a web service built to support biomedical data discovery and standardization by allowing users to describe their data using a JSON schema, regardless of the format and source of the data it describes. It can be used to connect data sources that have different data formats, and currently supports data formats such as CSV files, spreadsheets, relational databases, as well as objects in bucket storage. Data query functionality is provided through an API, which allows the standardized data to be searched using search terms, models, and data elements. In the case that data are not standardized, it also supports data search based on descriptions of the data itself.

Data Connect can also be useful to build data ingestion pipelines to acquire data from users and load it into the data lakehouse infrastructure. Specifically, the data organization and standardization API is recommended to standardize datasets before inserting them into the data Lakehouse. Additionally, once the data are standardized and data descriptors are kept in the data lake using a common JSON schema, the API can also be used to search for information across different datasets with different formats.

The Passport Broker is another solution currently developed by GA4GH to support user access control to different datasets in the repositories. The access control design paradigm is somewhat similar to a visa system employed by countries to allow international travel of different nationals. In the Passport Broker system, a visa can be issued to a user. The visa is made up of a unique visa ID, a name (the type of permission and access rights), an issuer ID, a general description, and the secret key given to the user to access the visa.

The Passport Broker’s API allows visas to be issued and changed, making it easy to manage access types to different datasets in the Lakehouse. The passport broker can be used to organize individual datasets in repositories according to their privacy and access requirements. This allows a system to issue a single passport to a user to grant access to datasets with valid visas within different repositories, instead of issuing multiple passports for each requested dataset. A list of users and their passport properties will have to be stored and managed by the system. Combined with Data Connect, the Passport Broker is a tool built to help with user access control across multiple datasets.

Lastly, LakeFS is an open-source solution built to provide a scalable and agnostic way to enable data version control using similar commands to Git that can be used to build data lake infrastructures (Hambardzumyan *et al.* 2022). LakeFS is a good candidate to integrate into the data lakehouse infrastructure since the practical git-like commands facilitate intuitive data management for users. Furthermore, it supports integration with Delta Lake and Ceph, as well as most object storage database systems supported by cloud providers.

Additionally, LakeFS can easily be integrated with most of the commonly used programming environments, such as Python, which is widely adopted by the Bioinformatics research community. LakeFS is format agnostic and therefore can be used to version control any dataset type. However, it should be noted that Delta Lake already offers data version management on the file level. Therefore, if Delta Lake is chosen as a storage framework, LakeFS’s versatile version control functionality is only necessary for highly complex data versioning use cases.

## 8 Discussion and conclusion

**Figure 4.**
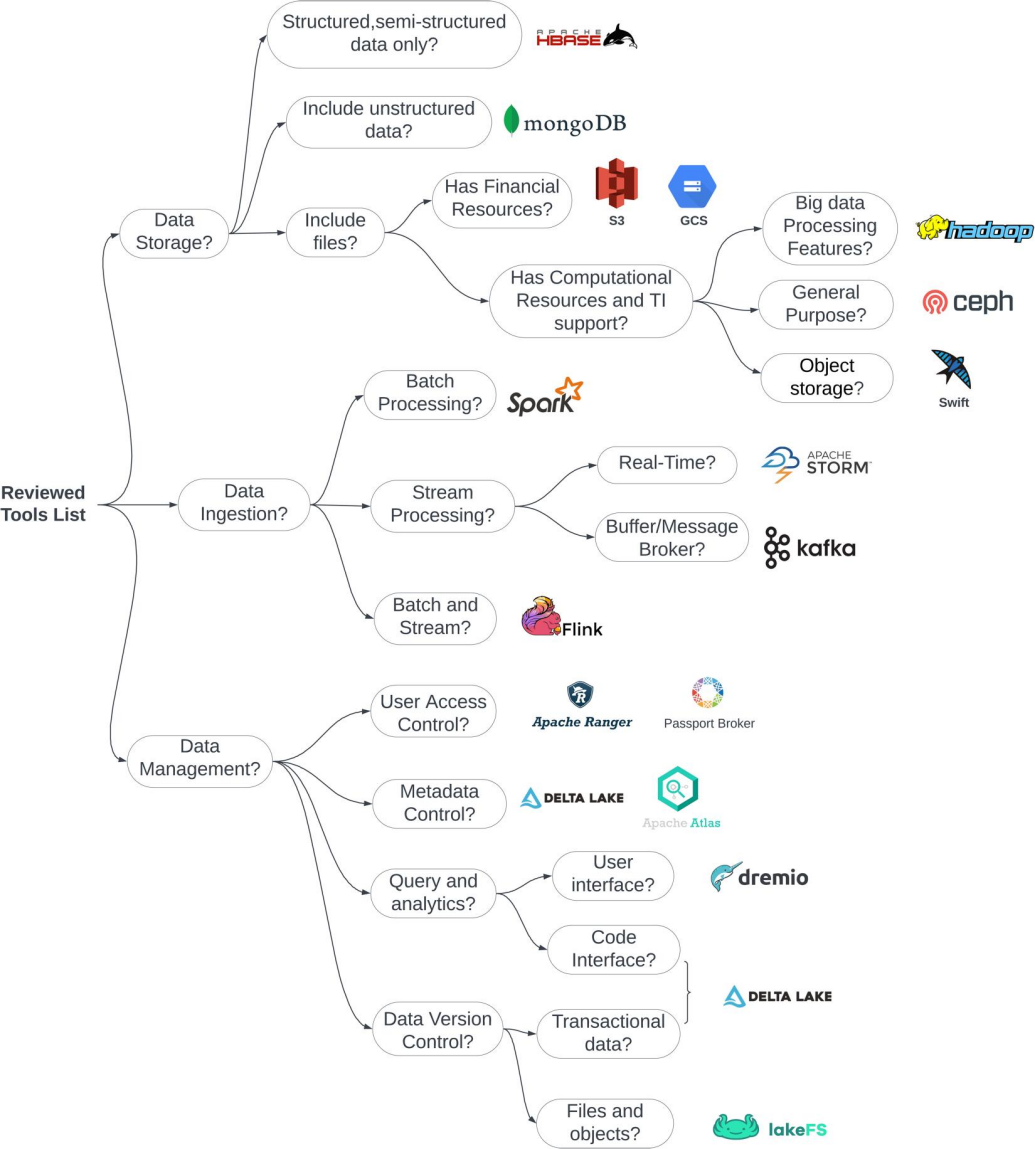
Decision tree for building data management infrastructure. The decision tree outlines tool selection based on category and features for building a data lakehouse. One tool may be enough for effective data storage, while data ingestion and management often need multiple tools. Apache Spark serves as a versatile processing option, but stream and batch data may require additional tools. A robust management setup could include LakeFS for versioning, Passport Broker for access control, and Delta Lake for metadata and analytics.

Building data management infrastructures for heterogeneous data in bioinformatics can be challenging due to the lack of available guidelines and recommendations. Research studies discussing the design of data management solutions can be found, but most present strategies apply only to specific types of data and use cases. Therefore, these studies do not address the challenges that arise with data management for very heterogeneous data. On the other hand, companies and studies in other fields of science have developed approaches to deal with heterogeneous data. These may be leveraged and combined with the specific strategies seen in relevant literature to help with the design process. By using the INFORM-Africa research hub as a use case, we could identify and address common data management challenges and software requirements (Fig. 2). These software requirements can be applied to multiple scenarios, combined with the different tools and strategies found to guide data infrastructure development processes (Fig. 4).

Up-to-date, accurate, and ready-to-consume data are invaluable for researchers and users requiring reliable information to conduct their research or to make good management decisions. Data management systems such as data lakes, data warehouses, or even non-relational databases may be sufficient for most use cases. However, for more complex use cases, a lakehouse architecture seems to be the most appropriate option due to its design paradigm that combines the best features of all these approaches. This is embodied through its flexible system design, which allows the storage of heterogeneous data as well as highly organized data used by end-user data consumers. This flexibility makes it more adaptable when project requirements change, which may arise as research projects expand, data volumes grow, and users’ needs also become more complex.

We also identified the importance of the FAIR4RS principles for the development of open, shareable, and accessible research software products. As mentioned, to make research software compliant with the FAIR4RS principles, it is necessary to ensure access via public software repositories, as well as to provide an open-source license and detailed documentation on building and using the software. Regarding data, the governance capabilities of lakehouse infrastructure inherently comply with the FAIR principles for data resources. By implementing practices such as effective metadata management, cataloging, and version control, you can ensure that your data are both findable within the repository and reusable. Additionally, incorporating data retrieval methods, such as APIs and software libraries, enables interoperability and integration with other tools.

Tools like Delta Lake and LakeFS can support the FAIRification process by managing metadata, controlling data versions, and providing users with transactional capabilities. Data catalogs and metadata management strategies can also be developed using custom APIs and non-relational databases. A custom API can be tailored for each project’s specificities, allowing it to collect, process, and transform data, extracting metadata or relevant information, and loading it into the database. For custom metadata management, flexible databases such as MongoDB are recommended for their scalability, performance, and ease of use and maintenance.

One key takeaway is the importance of a data federation approach. Storing data in a federated manner helps ensure compliance with data regulations that may vary by geographic location. Distributed storage tools and infrastructures are essential to implementing data federation. Tools such as Hadoop HDFS, Ceph, Swift, MongoDB, and HBase, which support distributed storage and processing, are particularly valuable in this context. All these tools offer access control in a certain capacity, but only Hadoop HDFS, OpenStack Swift, and MongoDB offer built-in data encryption capabilities that can be set up during the installation process. Swift’s and MongoDB’s encryptions are made via associated security engines and are provided at rest through capacity (disk storage), while Hadoop HDFS offers it natively and supports both rest through and in-transit (as it moves across a network of Hadoop clusters).

Despite the usefulness of open-source technologies for building comprehensive systems to manage heterogeneous data, none of them offers all the features needed to fulfill all the requirements to build an effective data management system. Therefore, to build a robust system without compromising security, usability, and maintenance, it is necessary to choose a suite of tools and combine them. Open-source storage solutions are typically not licensed with a fee, are customizable, and reusable, but often more difficult to implement and maintain. Setting up an infrastructure with the tools presented is not a trivial task, as it requires substantial computational resources, technical expertise, and ongoing support. Some organizations may need to maintain dedicated support teams to ensure continued uptime and reliability. This can pose a significant challenge for smaller or lower-resourced organizations. However, with careful planning, even these organizations can set up HDFS or non-relational database clusters and integrate them with Spark and Docker to simplify management and maintenance. Alternatively, cloud storage services provided by popular vendors such as Google Cloud and Amazon Web Services are not open source but are fully managed by the vendors, making them easier to maintain besides the low cost for operations with small volumes of data. For large volumes of data, costs can increase rapidly, posing a challenge for organizations with limited resources. Therefore, choosing between cloud services and open-source solutions depends on data volume, technical expertise, and support availability. For relatively low data volumes, cloud solutions may be more viable for resource-constrained organizations, as they require less effort and expertise to set up. However, for large data volumes, maintaining a cloud storage solution may be more costly than establishing and supporting an open-source infrastructure.

Besides the volume of data, costs associated with keeping a technical team, as well as the costs associated with server hardware are also relevant and should be considered. However, allowing federated approaches can be difficult since some countries do not host cloud data centers (cloud zones). Consequently, the data management systems need to be self-hosted at locations without cloud zones.

Open-source tools continue to be developed only if there is sufficient buy-in from users who contribute to their improvement. Therefore, it is important to choose tools that already have strong support from larger companies or the community, reducing the risk of relying on a tool that might become deprecated. For research organizations with limited financial resources, it is also essential to seek partners to share development and maintenance costs, helping to mitigate the risk of frameworks becoming obsolete or stagnating. For instance, the tools that are part of the Hadoop Ecosystem, MongoDB, and OpenStack are extremely well maintained and supported by companies and the community.

Although focused on a bioinformatics use case, the tools and solutions we presented can be applied to use cases in a variety of fields. For the bioinformatics data management infrastructure itself, the set of tools and technologies to consider can vary according to each project’s requirements and budget (Supplementary Fig. S1, available as supplementary data at *Bioinformatics Advances* online). In an ideal scenario where budget is not a limiting factor, full cloud solutions would be the preferred option due to their flexibility, scalability, and fully managed offerings that can be used for building data lakes and warehouses. If the goal is to build a fully open-source and self-managed solution, the Hadoop ecosystem can provide software alternatives to satisfy most project needs.

We recommend a combination of cloud-based solutions and self-managed, open-source solutions to build data lakehouses due to the lower price of cloud-hosted storage and the flexibility provided by open-source and self-hosted solutions to manage metadata. Furthermore, Fig. 4 presents a decision tree for the tool’s selection process based on key questions and features that different use cases can address for data storage and data ingestion. Cloud services such as Amazon S3 and Google Storage for object and file storage are great choices, which can be combined with systems such as Delta Lake to provide data management and dataset version control. These can be combined with tools from the Apache software suite to ingest and process data before storing it in cloud-hosted buckets.

## Supplementary Material

vbaf168_Supplementary_Data

## Data Availability

There are no new data associated with this article.
