# Supplementary material for: Review of open-source software for developing heterogeneous data management systems for bioinformatics applications
Source: Bioinform Adv. 2025 Jul 18;5(1):vbaf168. doi: 10.1093/bioadv/vbaf168 (PMC12321290; doi:10.1093/bioadv/vbaf168)
Supplement: vbaf168_Supplementary_Data [file vbaf168_supplementary_data.zip › Supplementary_Material.pdf]

Review of open-source software for developing heterogeneous data management systems for bioinformatics applications

Supplementary Material

Table S1. List of user requirements and their sub-requirements

Number	Description
1.	Dataset owners must be able to store heterogeneous raw data. More specifically,<br>A. Dataset owners must be able to specify in which geographic locations data is required to be hosted.<br>B. Dataset owners must be able to indicate which parts of datasets should be anonymized/de-identified.
2.	Dataset owners must be able to control access to datasets. More specifically,<br>A. Dataset owners must be able to authenticate with their credentials before being able to access data, make changes to a dataset, or change access privileges associated with a dataset.<br>B. Dataset owners must be able to control access privileges to a user or groups of users to a dataset.
3.	Dataset users, given appropriate access privileges, must be able to easily query and retrieve data. More specifically,<br>A. Users must be able to access a dataset through interfaces from typical data science tools.<br>B. Users should be able to search for, browse through, view, and access different datasets versions (data history).
4.	Given appropriate access privileges, users should be able to integrate different datasets stored on the infrastructure and perform their analysis in their own computational environments.

Table S2. List of software requirements and their sub-requirements

Number	Description
1.	The system must be able to store data organized in any type of schema. The system must therefore:<br>A. Support any data formats.<br>B. Be data schema agnostic.<br>C. Allow for high levels of data persistence.<br>D. Guarantee high availability.<br>E. Allow for distributed storage functionality.
2.	The system must be able to ingest data by supporting:<br>A. Ingestion pipelines/modules to collect data automatically from appropriate APIs.<br>B. Interface/API connectors to load data from a user.<br>C. Batch processing and real-time processing of data.<br>D. Data compression functionality.
3.	The system should be able to manage data and metadata by providing the following:<br>A. Access control over the raw data (restricted access).<br>B. Data anonymization functionality.<br>C. Data accessibility using SQL-like or any data science tool/programming language.<br>D. Data must be searchable (cataloged) and reusable.

E. Data must be cleaned after ingested.
F. Data version control (data history).
G. Connection interface.
H. Integrate with different tools.

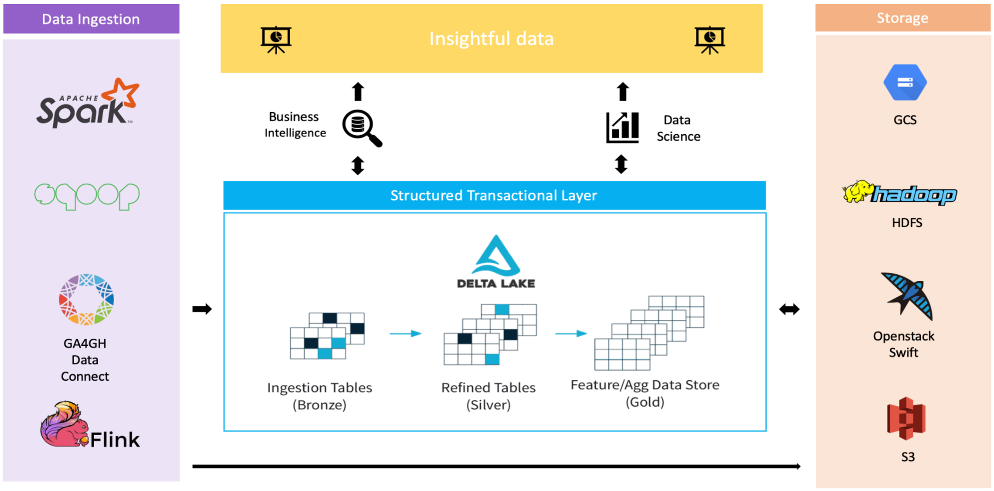

Figure S1. The proposed lakehouse infrastructure with associated tools. The data ingestion tools (left) can be chosen depending on users' needs. Apache Spark is a critical component since it allows for batch processing in ingestion pipelines as well as streamed data processing. If the application requires connection with biomedical databases, the GA4GH Data Connect software is the most promising tool to allow access to common biomedical databases. Flink is a good option if the infrastructure is mainly composed of Apache tools, as it can handle batch and real-time data. The Delta Lake is a promising tool to manage data for the transactional layer and enables data version control. For the storage layer, depending on the use case, cloud services such as Google Cloud Storage (GCS) and S3 may be the best option, however, Hadoop and Openstack Swift are good open-source framework alternatives.
